# Female detrusor underactivity: Two uterine leiomyoma cases

**DOI:** 10.1002/iju5.12223

**Published:** 2020-09-28

**Authors:** Ayami Shimizu, Ryuji Sakakibara, Maki Nakata, Naoki Takeshita, Akiko Takashima, Yosuke Aiba, Fuyuki Tateno, Hiroyoshi Suzuki, Masashi Yano

**Affiliations:** ^1^ Clinical Physiology Unit Sakura Medical Center Toho University Sakura Japan; ^2^ Neurology Division Department of Internal Medicine Sakura Medical Center Toho University Sakura Japan; ^3^ Department of Gynecology and Obstetrics Mitsui Memorial Hospital Tokyo Japan; ^4^ Department of Gynecology and Obstetrics Sakura Medical Center Toho University Sakura Japan; ^5^ Department of Urology Sakura Medical Center Toho University Sakura Japan

**Keywords:** detrusor underactivity, leiomyoma, urinary retention, urodynamics, uterus

## Abstract

**Introduction:**

Female urinary retention is rare.

**Case presentation:**

Case 1, a 35‐year‐old nulliparous woman, and case 2, a 47‐year‐old nulliparous woman, had transient urinary retention. A urodynamics revealed increased bladder sensation in case 1 and detrusor underactivity with a large post‐void residual in cases 1 and 2. Both women had a uterine leiomyoma of >10 cm in diameter. Soon after extraction of the tumor, retention episodes disappeared completely in case 1.

**Conclusion:**

Although rare, uterine leiomyoma should be listed as a cause of female detrusor underactivity.

**Abbreviations & Acronyms**
CIC: clean, intermittent self‐catheterization
EMG: electromyography
MRI: magnetic resonance imaging
Pabd: abdominal (rectal) pressure
Pdet: differential detrusor pressure (Pves − Pabd)
Pves: vesical (bladder) pressure
PVR: post‐void residual
UL: uterine leiomyoma

**Keynote message**

UL may cause urinary retention. We report on two cases with retention, in whom urodynamics revealed detrusor underactivity. Although rare, UL should be listed as a possible cause of female detrusor underactivity.

## Introduction

Only two urodynamics reports of UL^1^, ^2^, ^3^, ^4^, ^5^ have been published to date, in which Andrada *et al*.^6^ showed detrusor underactivity and sphincter EMG abnormality, and Huang *et al*.^7^ suggested outlet obstruction. We added two urodynamic cases similar to a case by Andrada *et al*.^6^

## Case presentation

### Case 1

**Fig. 1:**
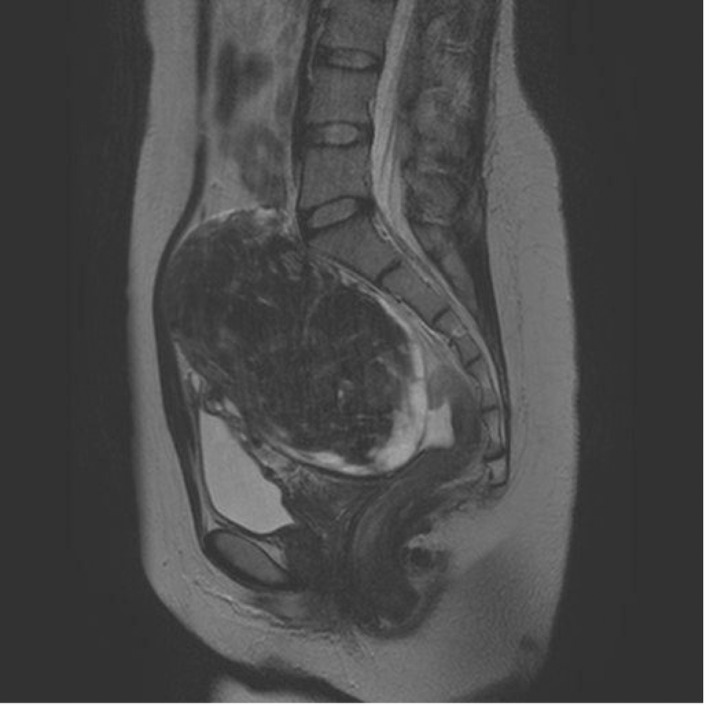
Abdominal MRI of case 1. An MRI scan revealed a UL 13 cm in diameter. Although the pelvic plexus itself could not be visualized, the size and the posterior‐base location of the tumor was thought to compress the pelvic plexus in this patient.

A 35‐year‐old nulliparous woman began to have transient acute urinary retention monthly at age 30 at the onset of her menstrual cycle. She visited a gynecology clinic and was found UL (details unknown), but without bladder management. Five years later she visited a urology clinic. She was catheterized of 830 mL PVR. Cystoscopy revealed no abnormalities. She was started on CIC three times a day with 30 mg/day urapidil (soon discontinued), which enabled her to void but still had voiding difficulty, which brought her to our clinic. In between retention episodes she had urinary urgency more than once a month; and voiding difficulty more than once a week. We did not perform a bladder diary. Blood test and urinalysis were normal. A MRI showed no abnormalities in the lumbar vertebra, but revealed a UL 13 cm in diameter (Fig. 1). Although the pelvic plexus could not be visualized, the size and the posterior‐base location of the tumor was thought to be enough to compress the pelvic plexus in this patient.

**Fig. 2:**
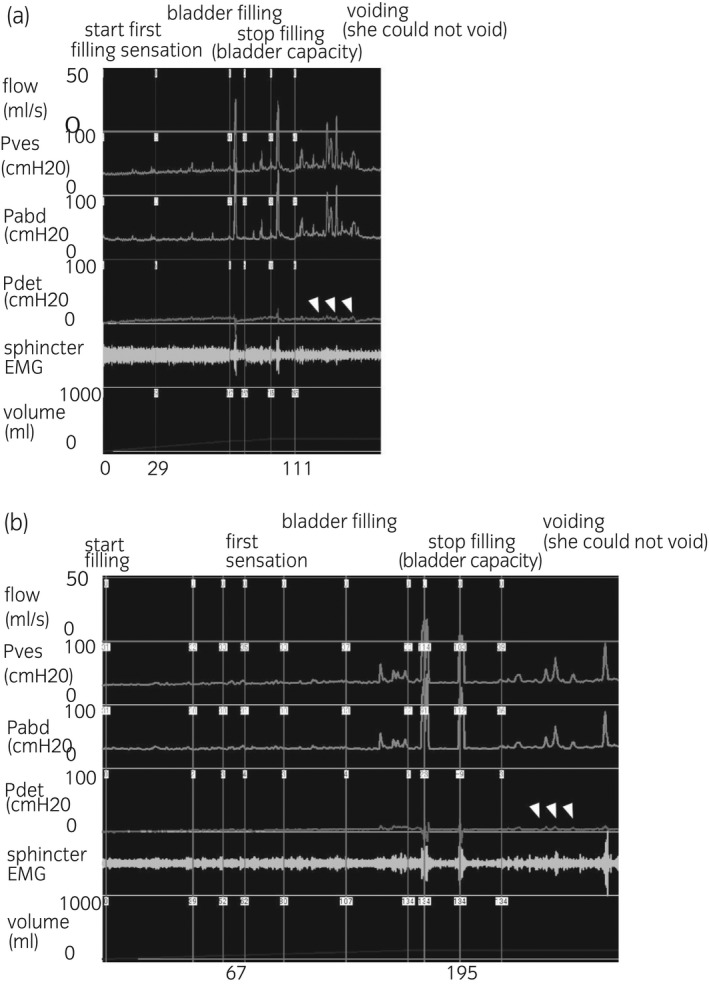
Urodynamic recording of case 1. (a) Initial urodynamic test: After evacuation, we started infusing saline into the bladder. The patient reported the first sensation at 29 mL (100 mL <normal <300 mL) and was found to have a bladder capacity of 110 mL (200 mL <normal <600 mL) but without detrusor overactivity (even after a provoked coughing maneuver) or low‐compliance detrusor, suggesting increased bladder sensation. Additionally, her sphincter EMG sound increased gradually, and no uninhibited sphincter relaxation was observed. We stopped infusion, and asked her to cough, but no leak was provoked. When we asked the patient to void, her sphincter EMG sound disappeared completely (however, the sphincter EMG recording was not good), and no unrelaxing sphincter (detrusor sphincter dyssynergia) was observed. She was unable to void and could not contract her bladder at all, even when straining. She had a PVR volume of 110 mL. No flow was obtained and pressure‐flow analysis could not be performed. Based on these results, she was diagnosed with detrusor underactivity. (b) Post‐surgical urodynamic test: During bladder filling, the patient reported the first sensation at 67 mL (100 mL <normal <300 mL) and was found to have a bladder capacity of 195 mL (200 ml <normal <600 mL), but without detrusor overactivity (even after a provoked coughing maneuver), indicating that increased bladder sensation remained, though it was less severe than in the initial test. When we asked the patient to void, her sphincter EMG sound disappeared completely (however, the sphincter EMG recording was not good). She was unable to contract her bladder at all, still suggesting detrusor underactivity. Flow, urinary flow.

An initial urodynamics (Fig. 2a) was performed 7 days after onset of urinary retention according to the standards of the International Continence Society method;^8^ a free uroflowmetry was not obtained; 80 min after voluntary urination, transurethral catheterization showed 30 mL of urine, indicating that she had no PVR in between transient retention episodes. During slow filling, she reported the first sensation at 29 mL and a bladder capacity of 110 mL but without detrusor overactivity. We asked the patient to cough, but no leak was provoked. When we asked her to void, she could not contract her bladder at all. She had a PVR volume of 110 mL. No flow was obtained and pressure‐flow analysis could not be performed. Based on these results, she was diagnosed with detrusor underactivity. A sphincter EMG and sacral reflexes were normal that excluded Fowler’s syndrome.

Soon after extraction of UL, the patient’s transient urinary retention disappeared completely, and she became able to void without PVR. A free uroflowmetry was still not obtained; 120 min after voluntary urination, transurethral catheterization showed 34 mL of urine. Bladder filling finding in a second urodynamics (Fig. 2b) performed 6 months after the surgery was almost the same with the prior test. She was still unable to contract her bladder on voiding.

### Case 2

**Fig. 3:**
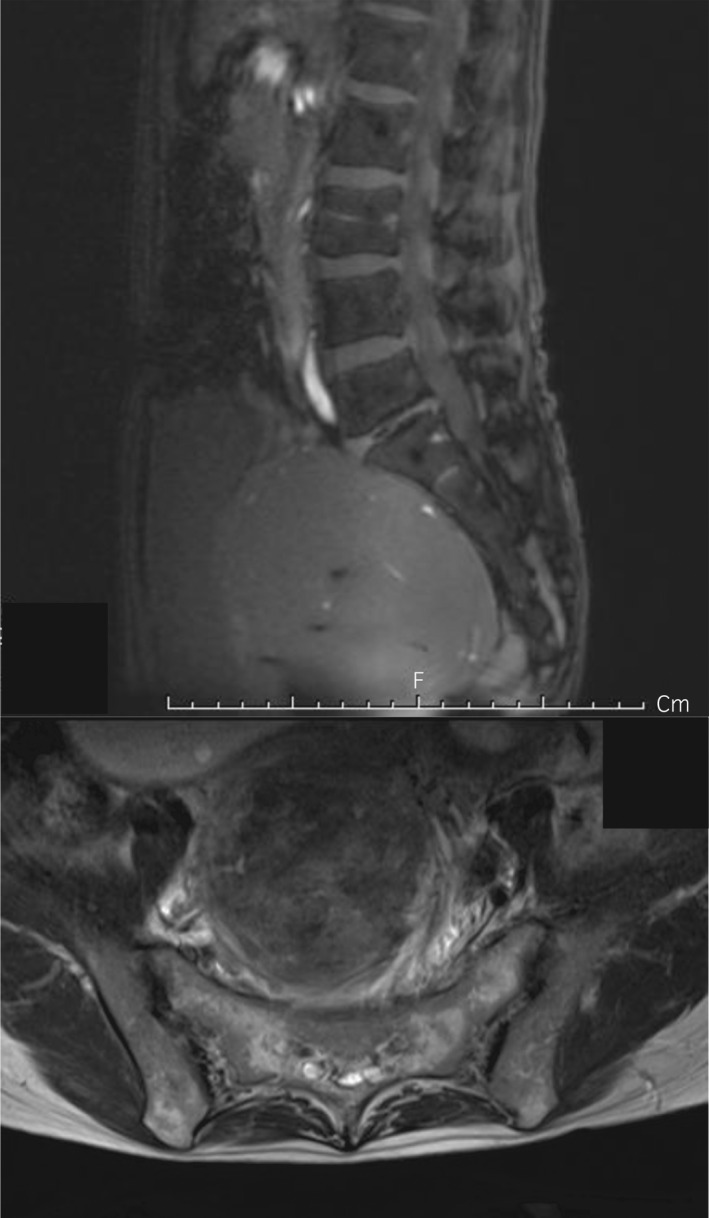
Abdominal MRI of case 2. An MRI scan revealed a UL 10 cm in diameter. Although the pelvic plexus itself could not be visualized, the size and the posterior‐base location of the tumor was thought to compress the pelvic plexus in this patient.

A 47‐year‐old nulliparous woman began to have transient acute retention at age 42, independent from her menstrual cycles. At age 38, she visited a gynecology health survey and was incidentally found to have UL (size unknown). She experienced episodes three times before being referred to us by a local urologist. On arrival, she was on 30 mg/day urapidil, and no CIC was performed. In between urinary retention, she had stress urinary incontinence (more than once a month); but no voiding difficulty. We did not perform a bladder diary. However, ultrasound echography revealed 68 mL of PVR. Blood test and urinalysis were normal. An MRI revealed UL 10 cm in diameter (Fig. 3). Although the pelvic plexus could not be visualized, the size and the posterior‐base location of the tumor was thought to be enough to compress the pelvic plexus in this patient.

A urodynamics was performed one month after onset of urinary retention, with discontinuation of urapidil three days prior. A free uroflowmetry was not obtained; 120 min after voluntary urination, transurethral catheterization showed 104 mL of urine. A urodynamics revealed normal storage findings. When we asked her to void, the patient could not contract her bladder at all. She had a large PVR volume of 600 mL. Based on these results, she was diagnosed with detrusor underactivity. Soon she was referred to another hospital to manage her UL (details not reported).

## Discussion

UL may cause either overactive bladder or retention.^2^, ^3^, ^4^, ^5^, ^6^, ^7^ We showed that UL caused detrusor underactivity in two cases, and both ameliorated after removal of UL (based on follow‐up findings in case 1). In most reports, direct compression of the bladder outlet by UL are postulated.^2^, ^3^, ^4^, ^5^, ^6^, ^7^ This might stem from the resemblance of UL to pelvic organ prolapse^9^ or leiomyoma of the bladder wall,^10^ in which benign outlet obstruction is suspected. In addition, Huang *et al*.^7^ urodynamically reported eight cases of UL. After recovery of acute urinary retention, a urodynamics showed a median urinary flow rate of 27 mL/min with preserved detrusor pressure of 28.5 cm H_2_O, suggesting outlet obstruction (nomogram and post‐void residual volume are not mentioned). In contrast, Andrada *et al*.^6^ demonstrated detrusor underactivity. Detrusor underactivity is described in lower neuron disorders (cauda equina/peripheral nerve lesions affecting the pelvic nerves) such as sacral herpes. In cases of leiomyoma, not only direct compression of the bladder neck but also pelvic plexus compression can occur. Although not observed in our cases, Andrada *et al*.^6^ reported sphincter EMG abnormality. This finding indicates that pelvic plexus compression may lead to pudendal nerve damage. Another urodynamic finding in the present case 1 was increased bladder sensation. Either decreased (complete lesion, i.e. electrophysiologically/pathologically “being cut” condition due to trauma, surgery etc.) or increased (incomplete lesion, i.e. “half‐damaged, half‐preserved” condition due to medical diseases etc.) bladder sensation can occur in afferent nerve damage.^11^ Diabetic neuropathy is a common cause of incomplete afferent nerve lesion. In patients with painful diabetic neuropathy, nociceptive microneurographic activity increases, accompanied by sensitization of the mechano‐insensitive C‐fibers.^12^, ^13^ Pelvic plexus compression might also bring about incomplete lesions of the afferent fibers.

Previous authors reported that urinary retention due to UL commonly occurs in early morning (early‐morning voiding difficulty or urinary retention).^7^, ^14^, ^15^ However, these findings were not obvious in our case. As for voiding‐phase symptom, it is a matter of debate that there is a huge discrepancy of the patients’ voiding pattern between urinary retention phase (large PVR/retention) and quasi‐normal phase (no PVR). We did not know the exact reason for the discrepancy. However, similar intermittent/transient worsening (retention) is observed during the day (in the morning, as mentioned above) and during a menstrual cycle (at the onset, in our case 1) in UL. These findings might well reflect the hormonal and circulatory/body‐position influences on the uterus.^7^, ^14^, ^15^ As for storage‐phase symptom, we did not perform a bladder diary; therefore we could not reproduce the storage‐phase urodynamic findings of increased bladder sensation by a bladder diary in case 1. In between transient retention, case 1 had only minimal storage symptom (urinary urgency more than once a month) by a questionnaire. Therefore, it is possible that increased bladder sensation could be a urodynamic artefact. Nevertheless, detrusor underactivity noted in UL, including ours, may shed light on the pathomechanisms of female retention that are often overlooked.

## Conclusion

We reported detrusor underactivity in two UL cases. Although rare, UL should be listed as a cause of female detrusor underactivity.

## Ethical approval statement

This study was approved by the Ethics Committee in Toho University.

## Author contributions

Ayami Shimizu contributed to acquisition of subjects and/or data, data analysis and interpretation. Ryuji Sakakibara contributed to study concept and design, acquisition of subjects and/or data, data analysis and interpretation, and preparation of manuscript. Maki Nakata contributed to critical review of the manuscript. Akiko Takashima, Naoki Takeshita, Yosuke Aiba, Fuyuki Tateno, Yuuka Sugisaki, Hiroyoshi Suzuki, and Masashi Yano contributed to acquisition of subjects and/or data.

## Conflict of interest

The authors declare no conflict of interest.
